# A hybrid energy-based and AI-based screening approach for the discovery of novel inhibitors of JAK3

**DOI:** 10.3389/fmed.2023.1182227

**Published:** 2023-10-10

**Authors:** Juying Wei, Youlu Pan, Zheyuan Shen, Liteng Shen, Lei Xu, Wenjuan Yu, Wenhai Huang

**Affiliations:** ^1^MDS Center, Department of Hematology, The First Affiliated Hospital, Zhejiang University School of Medicine, Hangzhou, Zhejiang, China; ^2^Key Laboratory of Neuropsychiatric Drug Research of Zhejiang Province, School of Pharmacy, Hangzhou Medical College, Hangzhou, Zhejiang, China; ^3^Hangzhou Institute of Innovative Medicine, Institute of Drug Discovery and Design, College of Pharmaceutical Sciences, Zhejiang University, Hangzhou, China; ^4^Institute of Bioinformatics and Medical Engineering, School of Electrical and Information Engineering, Jiangsu University of Technology, Changzhou, China

**Keywords:** JAK3, virtual screening, bioactivity evaluation, molecular dynamics, geometric deep learning

## Abstract

The JAKs protein family is composed of four isoforms, and JAK3 has been regarded as a druggable target for the development of drugs to treat various diseases, including hematologic tumors, cancer, and neuronal death. Therefore, the discovery of JAK3 inhibitors with novel scaffolds possesses the potential to provide additional options for drug development. This article presents a structure-based hybrid high-throughput virtual screening (HTVS) protocol as well as the DeepDock algorithm, which is based on geometric deep learning. These techniques were used to identify inhibitors of JAK3 with a novel sketch from a specific “In-house” database. Using molecular docking with varying precision, MM/GBSA, geometric deep learning scoring, and manual selection, 10 compounds were obtained for subsequent biological evaluation. One of these 10 compounds, compound **8**, was found to have inhibitory potency against JAK3 and the MOLM-16 cell line, providing a valuable lead compound for further development of JAK3 inhibitors. To gain a better understanding of the interaction between compound **8** and JAK3, molecular dynamics (MD) simulations were conducted to provide more details on the binding conformation of compound **8** with JAK3 to guide the subsequent structure optimization. In this article, we achieved compound **8** with a novel sketch possessing inhibitory bioactivity against JAK3, and it would provide an acceptable “hit” for further structure optimization and modification to develop JAK3 inhibitors.

## 1. Introduction

As a family of non-receptor tyrosine kinases, Janus kinases (JAKs) play a crucial role in type I and type II signal cascade pathways mediated by cytokines (1). Upon activation by binding with their specific ligands, JAKs undergo apposition and activation, leading to phosphorylation, dimerization, nuclear translocation, DNA binding, and target gene induction in the downstream signal transducer and activator of transcription (STAT) proteins (2). The activated JAK/STAT signal pathway is closely associated with inflammation, immune responses, and other physiological processes. Abnormal activation could lead to inflammatory diseases and cancer. Hence, JAKs have become an attractive target for the treatment of various clinical indications, such as autoimmune disease, myelofibrosis, tumors, and alopecia (3–5).

There are four members of the JAK family, namely JAK1, JAK2, JAK3, and tyrosine kinase 2 (Tyk2) (6). Of note, JAK3 is mainly expressed in hematopoietic cells and is involved in the γ-common chain (γc) to release the specific cytokines (i.e., IL-2, IL-4, IL-7, IL-9, IL15, and IL21) for the continuous development of T-cells, B-cells, and natural killer (NK) cells (7). By contrast, JAK1, JAK2, and Tyk2 are ubiquitous expressions compared with JAK3. It has been reported that JAK3 is a promising target for the treatment of hematological malignancies. For instance, the JAK3/STAT5b and JAK3/STAT6 cascade pathways play significant roles in leukemic B-cell precursors, and the overexpression of the JAK3 gene in B-lineage lymphoid malignancies has also been detected (8, 9). Meanwhile, the overexpression of mutated JAK3 has been confirmed in patients with acute T-cell lymphocytic leukemia, acute B-cell lymphocytic leukemia, acute myeloid leukemia, and lymphomas (10). Indeed, hematologic malignancies are among the most common cancers, and understanding their incidence and death is crucial for targeting prevention, clinical practice improvement, and appropriate research resources. Globally, incident cases of hematologic malignancies have been increasing since 1990, reaching 1,343.85 thousand in 2019. Of note, the age-standardized incidence rates (ASIR) for leukemia, multiple myeloma, non-Hodgkin, and Hodgkin lymphoma were 4.26, 1.42, 3.19, and 0.34 per 100,000 population in 2019, respectively (11).

**Figure 1:**
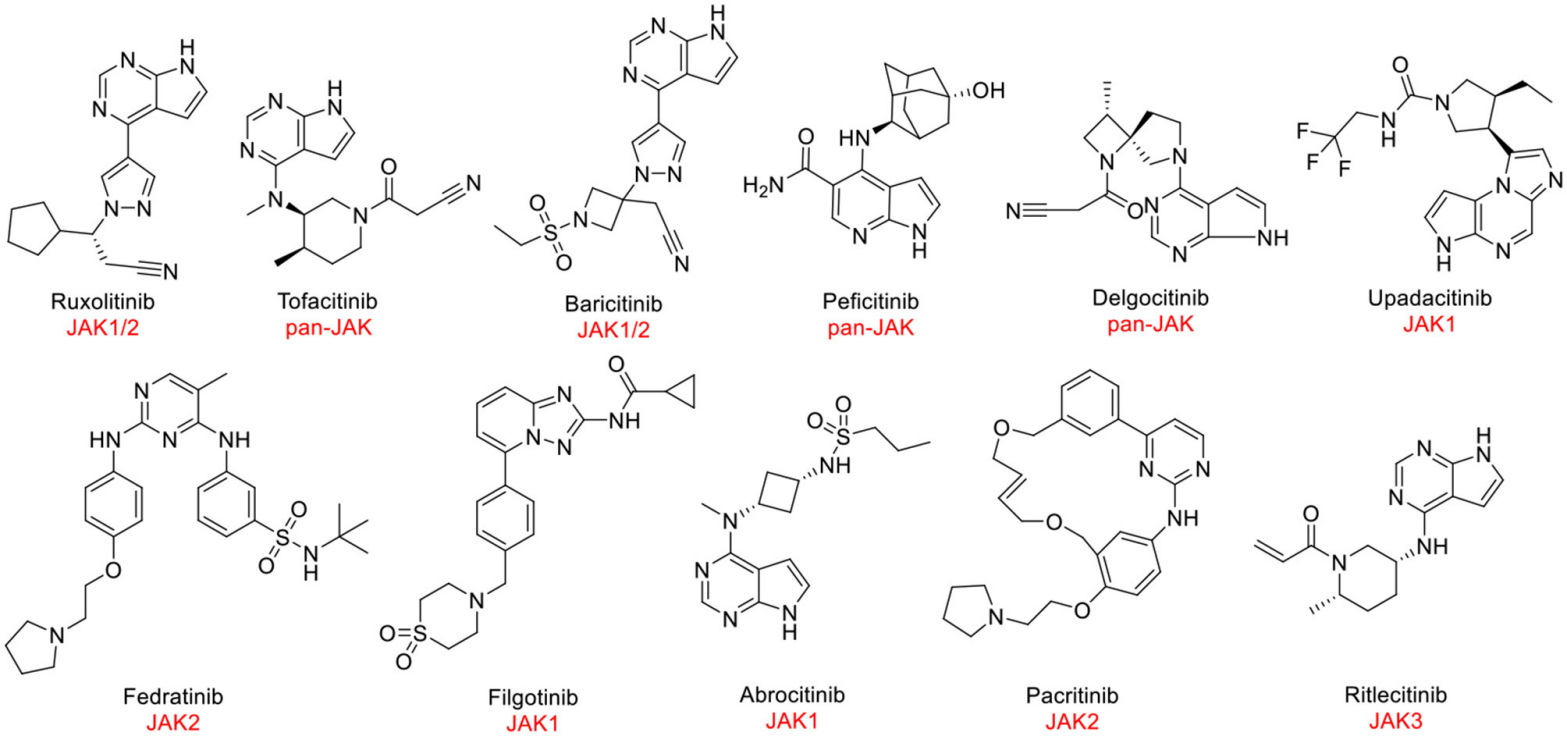
The structures of approved or clinically investigated JAK inhibitors.

Despite JAK3 exhibiting great capacity as a druggable target for hematological tumors, the discovery of JAK3 inhibitors remained an enormous challenge. At present, JAK1 and JAK2 antagonists have been approved by the Food and Drug Administration (FDA) and other regulatory agencies (Figure 1). Nevertheless, the majority of JAK3 inhibitors are still in clinical studies (6, 12), such as Ritlecitinib (Figure 1) in Phase III, which is being evaluated for dermatological disorders and other inflammatory diseases (13, 14). Therefore, it was meaningful to discover novel drug-like JAK3 inhibitors with specific sketches and explore their potential usage as therapeutic agents for hematological tumors.

For the discovery of new backbone JAK3 inhibitors, we employed computer-aided drug design approaches. Computational methods have been applied in the drug development process for screening and discovery of compounds with novel sketches in recent years (15, 16). Among them, virtual screening tools could significantly reduce the time and cost of drug discovery and increase development efficiency (17). In the meantime, molecular dynamics (MD) simulation would accurately assess the interaction between active molecules and targets (18). We combined these two methods and constructed a computational screening protocol to identify novel compounds as potential JAK3 inhibitors. Initially, an “*In-house*” molecule library was well prepared. Subsequently, different precision molecular docking methods were used for further screening. The molecules were filtered using DeepDock, a geometric deep learning algorithm, and combined with manual selection to achieve the final molecules. Ultimately, we identified one JAK3 inhibitor (compound **8**) with good inhibitory activity, exhibiting significant structural differences in the backbone with existing JAK3 inhibitors. Additionally, it also provided an acceptable “hit” for further optimization or modification to discover novel active compounds targeting JAK3. Meanwhile, the screening strategy in this article also provided a reliable reference for the development of active compounds with other targets.

## 2. Material and methods

### 2.1. Protein and ligand preparation

The x-ray co-crystallized protein structure (PDB ID: 5LWN) was acquired from the RCSB protein database for further protein preparation. A total of 110 JAK3 protein structures were recorded in the RCSB database. After filtering by species and non-covalent ligand, we finally selected 5LWN (1.6 Å) as our target for performing virtual screening. Protein preparation, including preprocessing, H-bond assignment, and restrained minimization, was performed through the Protein Preparation Wizard module (19) in the Schrödinger 2021-2 suite. In the preprocessing step, any issues with the protein were identified and resolved using default parameters by filling in the missing side chains and loops. Henceforth, the optimization of hydrogen bond assignments was implemented by sampling water orientations and using PROPKA at pH 7.4. Ultimately, it was followed by restrained energy minimization by adopting the OPLS4 force field to converge heavy atoms to an RMSD of 0.30 Å.

### 2.2. Database preparation

Two components, namely the commercial compound database ChemDiv and MCE's drug-like compound database, constituted the “*In-house*” database applied for screening. All ligands were performed by default parameters through the LigPrep module (Schrödinger 2012-2 suite). Hydrogenation, salt removal, tautomer generation, and ionization state calculation using the Epik (version 5.6) program at pH 7.0 ± 2.0 were carried out under the OPLS4 force field. Furthermore, up to 32 stereoisomers can be generated for each ligand under the computational condition of retaining specific chirality.

### 2.3. Receptor grid generation

For the next docking step, the corresponding file was created based on the center coordinates of the protein pocket. The center coordinates of the original ligand were defined as the center coordinates for the docking process, and the original ligand was removed to generate the corresponding grid file. These procedures were utilized by the receptor grid generation module of the Schrödinger 2012-2 suite.

### 2.4. Docking-based virtual screening

In this article, this specific virtual screening workflow mainly contained a continuous filtering procedure with different precisions for SP, XP, and MM/GBSA. Initially, based on the “*In-house*” database screening and the performance of the SP precision of the Glide dock, the top 250,000 molecules were chosen for scoring. Subsequently, 10 docking conformations of each compound were set for the next round of screening through the XP precision of Glide docking. In the third round, 20,000 molecules gained from the Glide docking were further filtered using MM/GBSA to obtain molecules, which were scored by applying the DeepDock approach combined with manual selection. Finally, 10 molecules were selected for further bioactivity evaluation.

### 2.5. Glide docking

The Glide molecular docking module of Schrödinger's kit is a powerful tool widely used in the field of computational drug discovery. It enables the prediction of the three-dimensional structure of a small molecule in a complex with a protein target, which is crucial for understanding the molecular interactions between a drug candidate and its target protein. These interactions ultimately determine the efficacy and specificity of the drug. The Glide module employs a combination of sophisticated force fields and scoring functions to model the binding of the small molecule to the protein target, considering various physical and chemical properties such as shape complementarity, hydrogen bonding, and electrostatic interactions.

In this experiment, both the SP precision and XP precision of the Glide docking modules were used in the same manner. First, the pre-prepared receptor grid file of the JAK3 target and the ligand file were loaded into the system. The force field was set to OPLS4, and the desired precision level was selected, which determines the accuracy of the simulation. To achieve optimal results, the maximum number of output structures and the number of poses per ligand to be included were all set to 5. Finally, the docking simulation was initiated by running the program.

### 2.6. MM/GBSA approach

Molecular mechanics/generalized Born surface area (MM/GBSA) is a computational method used to estimate the binding free energy of a molecule in a protein-ligand complex. The method is used in computational drug discovery to predict the binding affinity of a small molecule to a protein target. The MM/GBSA approach combines the strengths of molecular mechanics and generalized Born methods to provide an accurate and efficient prediction of binding free energy.

The binding free energy change was calculated using the MM/GBSA calculation method. The docked complexes of JAK3 with different ligands were minimized using the local optimization feature in Prime Wizard of Maestro (20). The OPLS4 force field was utilized to determine the binding energy for a set of receptors and ligands. The following equation was employed to calculate the binding free energy:

$$\begin{array}{l} \Delta \text{G}\left(\text{bind}\right)=\Delta \text{G}\left(\text{solv}\right)+\Delta \text{E}\left(\text{MM}\right)+\Delta \text{G}\left(\text{SA}\right) \end{array}$$

ΔE(MM) represents the difference between the minimized energies of the protein-ligand complexes and the sum of the energies of the unbinding protein and inhibitor. ΔG(Solv) represents the variance between the GBSA solvation energy of the protein-ligand complexes and the sum of the solvation energies for the protein and ligand. ΔG(SA) encompasses some of the surface area energies in the protein and ligand and the difference in the surface area energies for the complexes. The minimization of the docked complexes was carried out using a local optimization feature of the Prime.

### 2.7. DeepDock

The DeepDock program on GitHub is a deep learning framework for protein-ligand docking, a computational technique used to predict the binding affinity between a protein and a small molecule. The program uses a convolutional neural network (CNN) to learn the relationship between a protein and ligand's three-dimensional structures and their corresponding binding affinity. To begin, we applied the MASIF algorithm to determine the interfacial characteristics of JAK3 when bound to a molecule. The model then processes 2D molecular and protein pocket graphs as input and derives continuous representations. Using these representations, it calculates a statistical potential based on the likelihood of distances between protein-molecule pairs. Finally, it uses an optimization technique to produce the binding configuration of the molecule.

### 2.8. Induced fit docking

Induced fit docking (IFD) was applied for consistent poses of compound **8**. Standard protocol was employed to generate up to 20 poses by utilizing the above receptor grid and ligand sampled according to the conformations of rings. After that, the side chains of the protein were trimmed based on the B-factor before docking, and these residues were restored to their original form in the prime refinement stage. Ultimately, glide redocking was adopted to generate a small number of additional conformations within 30.0 kcal/mol of the best structure to improve the final pose.

### 2.9. Inhibition rate and IC_50_ assay

The tested compounds were dissolved in DMSO to make a 10 mM stock solution, which was further diluted to a drug solution of 25 μM. Initially, 2× ATP and substrate solution and 2× kinase and metal solution were prepared using assay buffer (MgCl_2_ 2 mM, MnCl_2_ 1 mM, SEB 12.5 nM, and DTT 0.5 mM). From each well of the 96-well plate, 25 μl of the drug solution was taken and then transferred to a 384-well plate, which was provided with two duplicate wells. Then, 2.5 μl of 2× kinase and metal solution was mixed and incubated in a polystyrene-coated 384 assay plate for 10 min at 25°C. 2× XL665 and antibody solution were prepared using a detection buffer. A volume of 5 μl of kinase detection reagent was added to the well and incubated for 60 min at 25°C. The fluorescence signals of 620 nm (Cryptate) and 665 nm (XL665) were read using a microtiter plate reader. Staurosporine is a positive control compound and is a prototypical ATP-competitive kinase inhibitor in that it binds to many kinases with high affinity. Its main biological activity is the inhibition of protein kinases through the prevention of ATP binding to the kinase (21). The experiment was performed in parallel three times, and the average value was taken.

Initially, the tested compound was dissolved in DMSO to create a 10-mM stock solution, which was further diluted to a drug solution with 50× test concentrations for later use. The test concentrations were reached through dilution at a 10-fold gradient and were 0.1 nM, 1 nM, 10 nM, 100 nM, 1,000 nM, and 10,000 nM, respectively. First, 2× ATP and substrate solution and 2× kinase and metal solution were prepared using assay buffer (MgCl_2_ 2 mM, MnCl_2_ 1 mM, SEB 12.5 nM, and DTT 0.5 mM). From each well of the 96-well plate, 25 μl of the drug solution was taken and then transferred to a 384-well plate, which was provided with two duplicate wells. Then, 2.5 μl of 2× kinase and metal solution was mixed and incubated in a polystyrene-coated 384 assay plate for 10 min at 25°C. 2× XL665 and antibody solution were prepared using a detection buffer. A volume of 5 μl of kinase detection reagent was added to the well, and incubated for 60 min at 25°C. The fluorescence signals of 620 nm (Cryptate) and 665 nm (XL665) were read using the microtiter-plate reader. Finally, the IC_50_ value of JAK3 kinase was calculated using the equations. In this assessment, tofacitinib was applied as the reference compound and dissolved in DMSO. Tofacitinib is a JAK inhibitor on the market, discovered and developed by the National Institutes of Health and Pfizer. The experiment was performed in parallel twice, and the average value was taken.


$$\begin{array}{l} \text{Y}=\text{Bottom}\\ +\left(\text{Top}-\text{Bottom}\right)/\left(1+10^{\wedge }\left(\left(\text{LogI}{\text{C}}_{50}-\text{X}\right)\times \text{hillslope}\right)\right) \end{array}$$

### 2.10. CCK-8 assay

Cell culture. The MOLM-16 cells were purchased from ATCC and cultured in RPMI-1640 medium (ATCC) with FBS (Gibco) and penicillin-streptomycin. All cells were maintained in a complete medium at 37°C with 5% CO_2_.

Cell growth inhibition was tested by applying the enhanced cell counting kit-8 (CCK-8, Beyotime) assay. Initially, the MOLM-16 cells were counted, and approximately 10,000 cells per well were seeded in a 96-well cell culture plate (Corning Inc.). Subsequently, after incubation at 37°C in a humidified atmosphere with 5% CO_2_ for 24 h, the culture medium was replaced by a series of concentrations of compound 8 diluted with the corresponding culture fluid. Three replicates were made for each measurement. After co-incubation for 72 h, 10 μl of CCK-8 reagent was added into each well, and OD at 450 nm was measured using a multifunction microplate reader after incubation for 1 h at 37°C. The percentage of each concentration that accounted for the control was presented as cell viability. The IC50 value was calculated using SPSS. The experiment was performed in parallel three times, and the average value was taken.

### 2.11. Molecular dynamics

The 10 molecules selected for complex conformations with JAK3 underwent MD simulation studies. These molecules were then processed through the protein preparation wizard module. The input system was constructed using the System Builder module of the Schrödinger 2012-2 Suite. Under the default parameters, the complex molecule was placed in the center of a box filled with SPC water molecules. The ensemble class for molecular dynamics simulations was set to NPT. The temperature was set at 300 K. The boundaries of the box were established at a distance of 10 Å from the farthest radius of the protein. Notably, 0.15 M NaCl and small quantities of Na+ and Cl- were filled in to balance the system charge. The prepared model was loaded into the MD module for further simulation work. The duration of the simulation was set to 1,000 ns, and the recording interval was set to 1 ns for each recording. Finally, the dynamics simulation process was performed under a 300 K temperature and a 1.01325 bar pressure. The results of the simulations were analyzed using simulation interaction analysis.

## 3. Results

### 3.1. The preparation of protein

In the beginning, the protein structure of JAK3 was obtained from the Protein Data Bank (PDB) (PDB ID: 5LWN, https://www.rcsb.org/). Using the Schrödinger 2021-2 suite's Protein Preparation Workflow, the protein was prepared for further analysis. Based on the default parameters, structural repair, hydrogenation protonation, hydrogen bond optimization, and energy minimization were performed for the JAK3 protein.

### 3.2. “*In-house*” database construction

The molecular diversity in the molecular library has been proven to be a vital factor in ensuring a high hit rate for virtual screening. To enhance this diversity, we constructed an “*In-house*” database by combining the ChemDiv compound library and the drug-like molecule library from MCE. To further optimize this database, we utilized the LigPrep module from the Schrödinger 2021-2 suite to prepare it for future applications. This preparation step generates the three-dimensional structure and ensures the molecules have the correct protonation state (19).

### 3.3. Hybrid virtual screening workflow

**Figure 2:**
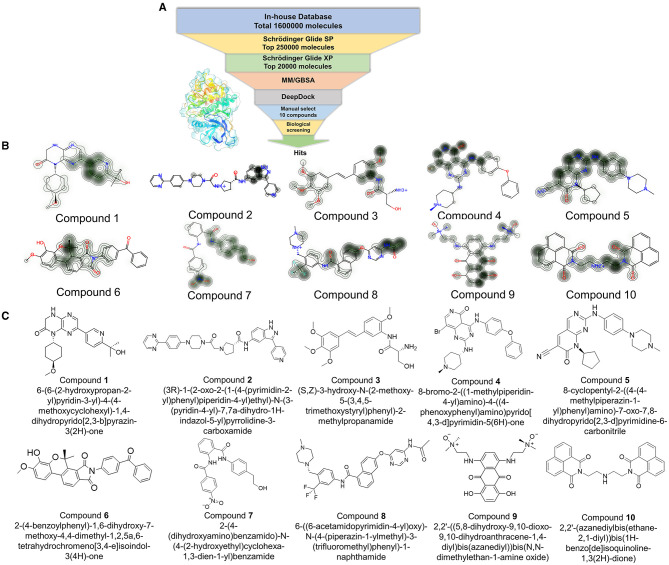
**(A)** The workflow of virtual screening; Glide_SP: Glide extra precision (SP); Glide_XP: Glide extra precision (XP); MM/GBSA: molecular mechanics with generalized born and surface area solvation. **(B)** The contribution of each atom was shown using a contour line in each compound. **(C)** The original structures of the selected 10 compounds.

Based on the resolved co-crystal structure of JAK3, high-throughput virtual screening (HTVS) was conducted to find the specific molecules based on the “*In-house*” database. The hybrid virtual screening workflow consisted of the following components, including Glide_SP and XP screening methods with two different accuracies, as well as MM/GBSA solvation scoring and DeepDock algorithms. The purpose of assembling such a hybrid screening workflow is to improve the precision of the final virtual screen and to better filter out molecules with no inhibitory activity. This self-built database contained 1.6 million small molecules and was used as a screening library reference. The virtual screening workflow and the corresponding results are demonstrated in Figure 2 and Table 1, respectively. Notably, OPLS4 was applied as the force field during the calculation.

**Table 1:** The docking score of selected 10 small molecules.

**Entry**	**Force field**	**Docking score (SP) (kcal/mol)**	**Docking score (XP) (kcal/mol)**	**MM–GBSA dG bind (kcal/mol)**	**DeepDock score**	**Inhibit rate (%)**
Compound 1	OPLS4	−9.978	−10.816	−77.05	−67.82	15.60 ± 0.68
Compound 2	OPLS4	−8.829	−8.583	−61.57	−98.95	44.50 ± 1.43
Compound 3	OPLS4	−8.491	−8.618	−72.72	−73.01	16.58 ± 2.18
Compound 4	OPLS4	−9.401	−9.756	−75.58	−115.33	93.37 ± 0.01
Compound 5	OPLS4	−8.821	−8.067	−71.47	−179.63	75.22 ± 0.85
Compound 6	OPLS4	−8.474	−8.114	−83.41	−82.11	27.94 ± 0.79
Compound 7	OPLS4	−8.374	−8.232	−76.62	−112.38	17.53 ± 1.63
Compound 8	OPLS4	−8.154	−8.079	−76.45	−160.79	83.46 ± 0.11
Compound 9	OPLS4	−8.350	−8.617	−68.84	−106.46	35.15 ± 1.61
Compound 10	OPLS4	−7.870	−8.419	−67.72	−85.46	25.56 ± 0.85

First, the molecules in the “*In-house*” database were screened using the Glide_SP module (22–24). After screening, the 250,000 molecules with scores ≤-7.068 kcal/mol were chosen, and a more precise screen was further conducted using the Glide_XP module, which used a more complex scoring function than the Glide_SP score, with more restrictive restrictions on ligand-receptor shape complementarity.

Subsequently, 20,000 molecules obtained from the Glide_XP stage with scores ≤-8.030 kcal/mol were subjected to the MM/GBSA calculation (25). A geometric deep learning framework algorithm “DeepDock” was further applied to evaluate the contribution of different atoms to these molecules for target binding to assist in the subsequent manual selection. Finally, 10 compounds were acquired from the rest of the 5,000 molecules. Noteworthily, the selecting method included comprehensive consideration of the scoring and interactions to remove the wrong binding mode molecules. On the other hand, through the experience of medicinal chemists, the molecules possessing higher binding affinity were estimated, which possibly excluded some molecules that were not drug-like. In Figures 2B, C, the corresponding structures of 10 selected compounds based on our workflow were exhibited.

### 3.4. Inhibitory activity assay

**Figure 3:**
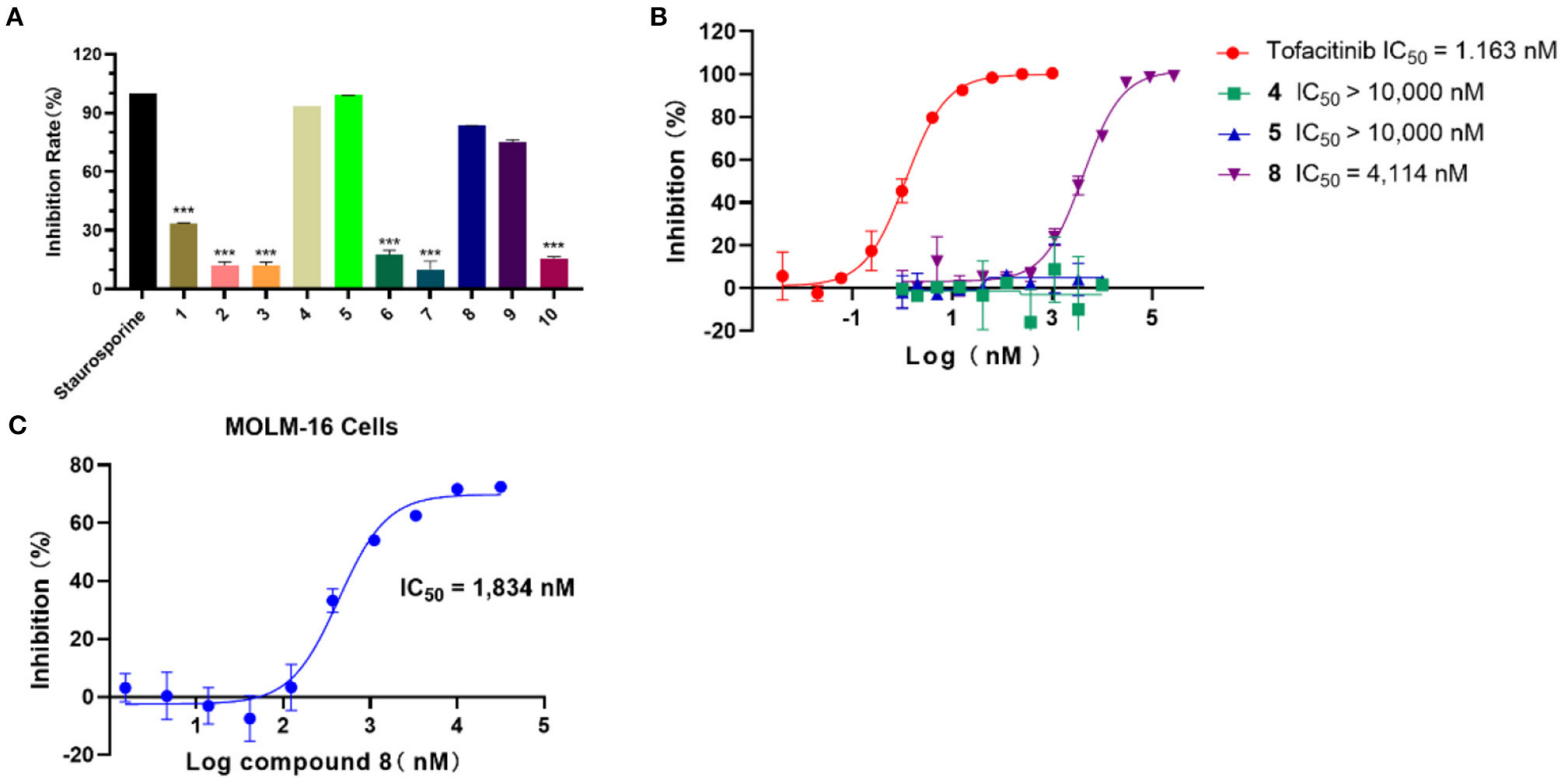
**(A)** The inhibition rate (%) assay of 10 selected compounds against JAK3 (staurosporine was the reference compound), *n* = 3. **(B)** The IC_50_ evaluation of selected compounds (compounds **4**, **5**, and **8**), n = 2; IC_50_: half maximal inhibitory concentration. **(C)** The anti-proliferation assay of compound **8** against the MOLM-16 cell line, n = 3; MOLM-16 cell line: megakaryoblastic leukemia cell line.

In light of the HTVS results, the inhibition activity assay was conducted for the selected 10 compounds (25 μM). As shown in Figure 3A, compounds **4**, **5**, and **8** exhibited a higher inhibition rate compared with other compounds. According to the experimental results, the IC_50_ values of compounds **4**, **5**, and **8** against JAK3 kinase were further evaluated (Figure 3B), and compound **8** showed inhibitory activity against JAK3 with an IC_50_ value of 4,114 nM. Moreover, the anti-proliferation test of compound **8** against hematologic cancer cells, like the megakaryoblastic leukemia cell line (MOLM-16), was carried out by applying cell counting kit-8 (CCK-8) (6). As demonstrated in Figure 3C, the IC_50_ value was reached at 1,834 nM for the MOLM-16 cell line. Initially, compound **8** was reported as a type II CDK2 inhibitor in the clinical study. Herein, we discovered that this compound also exhibited bioactivity against JAK3. The combined activity assessment demonstrated the reliability of our HTVS workflow, and it also provided a favorable hit for further structure optimization to develop JAK3 inhibitors.

### 3.5. Induce fit docking

**Figure 4:**
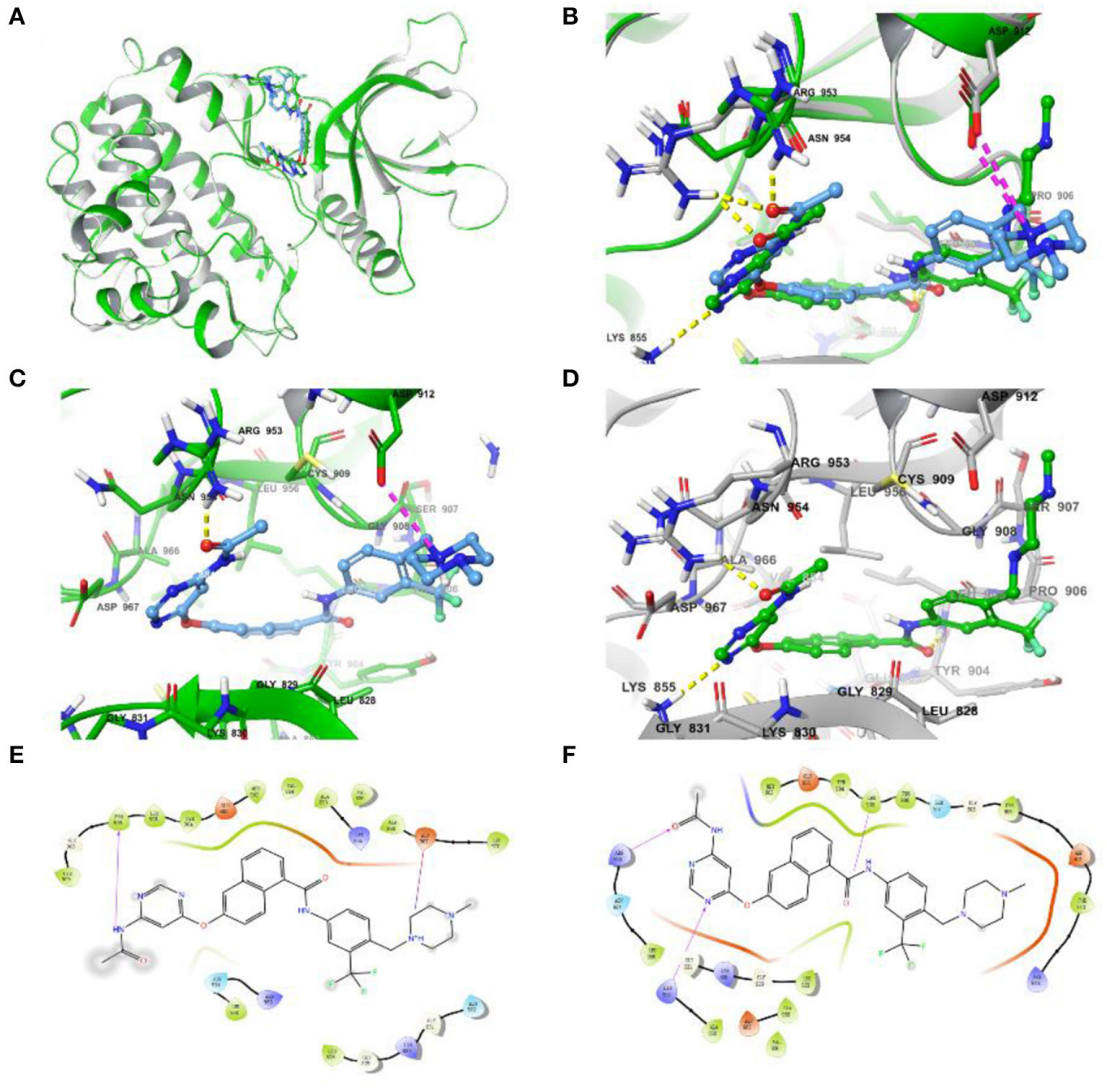
**(A)** Differences in complex conformation before and after induced-fit docking. **(B)** Conformational variations of key amino acid residues (the yellow dashed line represents the H-bond interaction force, and the purple dashed line represents the salt bridge) in the binding pocket before and after induced fit docking. **(C)** The interactions of JAK3 and compound 8 before induced fit docking. **(D)** The interactions of JAK3 and compound 8 after induced fit docking. **(E)** The 2D diagram of protein-ligand interaction before induced fit docking. **(F)** The 2D diagram of protein-ligand interaction after induced fit docking.

Due to the Glide SP/XP docking module, without taking into account the flexibility of the protein, the assessment of the effect on side-chain conformation change and backbone movement of the protein was further performed before MD simulation via an induced-fit docking approach (Figure 4). As shown in Figures 4A, B, the conformation displayed slight differences and also demonstrated the necessity of induced-fit docking. Furthermore, the more reasonable binding conformation of compound **8** with JAK3 was obtained, and additional H-bond and hydrophobic interactions were observed after induced-fit docking (Figures 4C–F). Finally, a more stable and rational binding mode was achieved for further MD simulation. Compared with Figures 4E, F, compound **8** could generate additional H-bond interactions with Lys855 and Arg953 after induced-fit docking.

### 3.6. Molecular dynamics and binding mode analysis of compound 8

**Figure 5:**
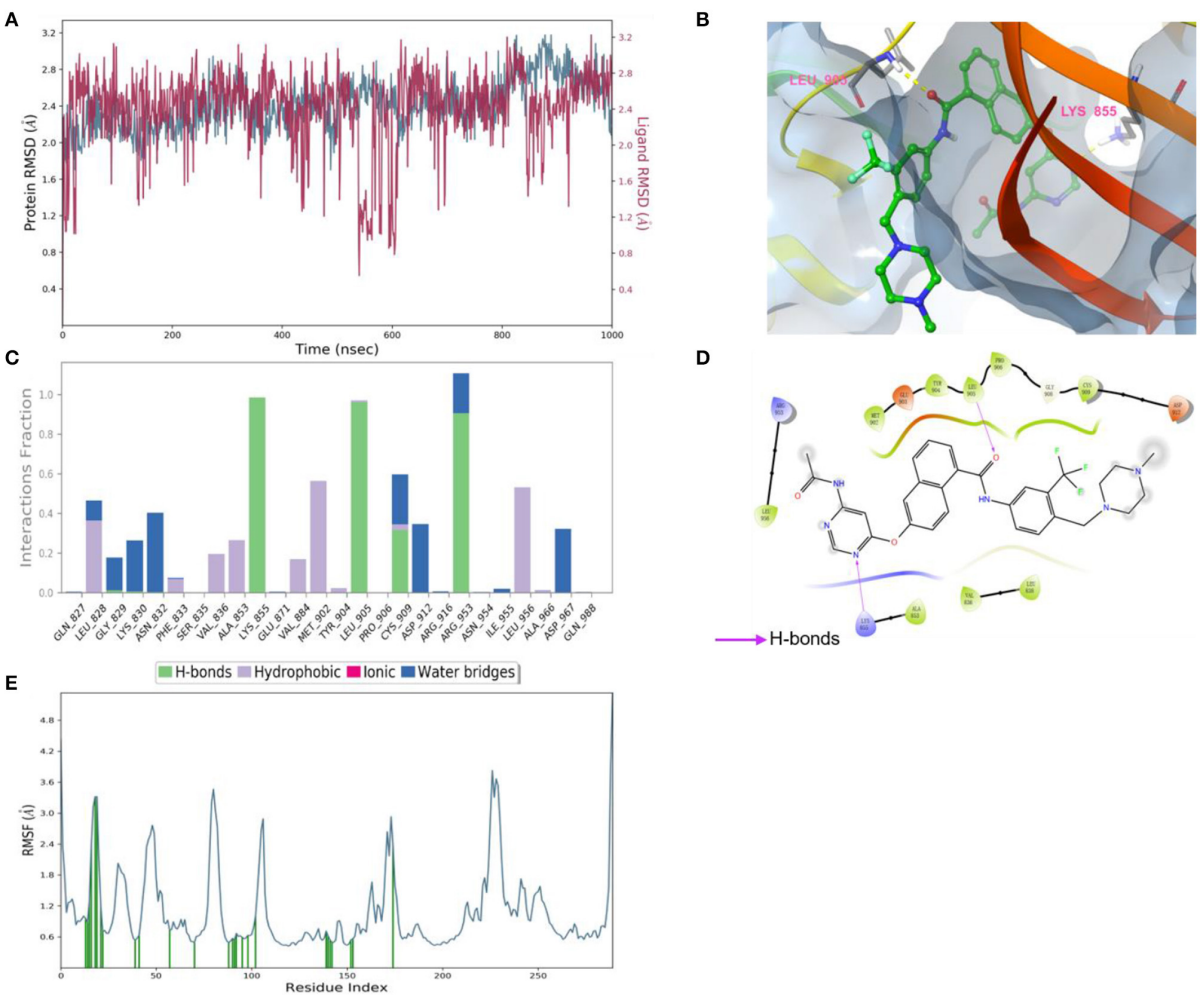
**(A)** Root-mean-square error (RMSD) value of compound **8** (red) and JAK3 (blue). **(B)** The interactions of JAK3 with compound **8**. The yellow dashed lines represented H-bonds. **(C)** The contribution of the compound **8**—JAK3 complex. **(D)** The 2D diagram of protein-ligand interaction. The pink arrows represented H-bond interactions. **(E)** The root-mean-square fluctuation (RMSF) measured the average deviation of the residue of the JAK3 protein.

According to the results of bioactivity evaluation and induced-fit docking, the MD simulation of compound **8** with JAK3 was carried out using the Schrödinger 2021-2 suite (26, 27). As displayed in Figure 5A, the MD simulation was conducted for 1,000 ns, and the binding complex was in equilibrium. During the last 100 ns, the protein and ligand achieved stable status. Subsequently, the last conformation was chosen for further analysis. The N atom of the pyrimidine moiety could generate a strong H-bond interaction with Lys855. Besides, the O atom of amide formed an additional H-bond interaction with Leu905 (Figure 5B). Meanwhile, the –CF_3_ of compound **8** could be inserted into a specific hydrophobic pocket surrounded by the side chains of Leu905, Gly906, and Arg953 to form strong hydrophobic interactions (Figures 5C, D). These specific interactions made compound **8** possess inhibitory activity against JAK3.

### 3.7. MD simulation analysis insights into the effective inhibition of JAK3 inhibitors

**Figure 6:**
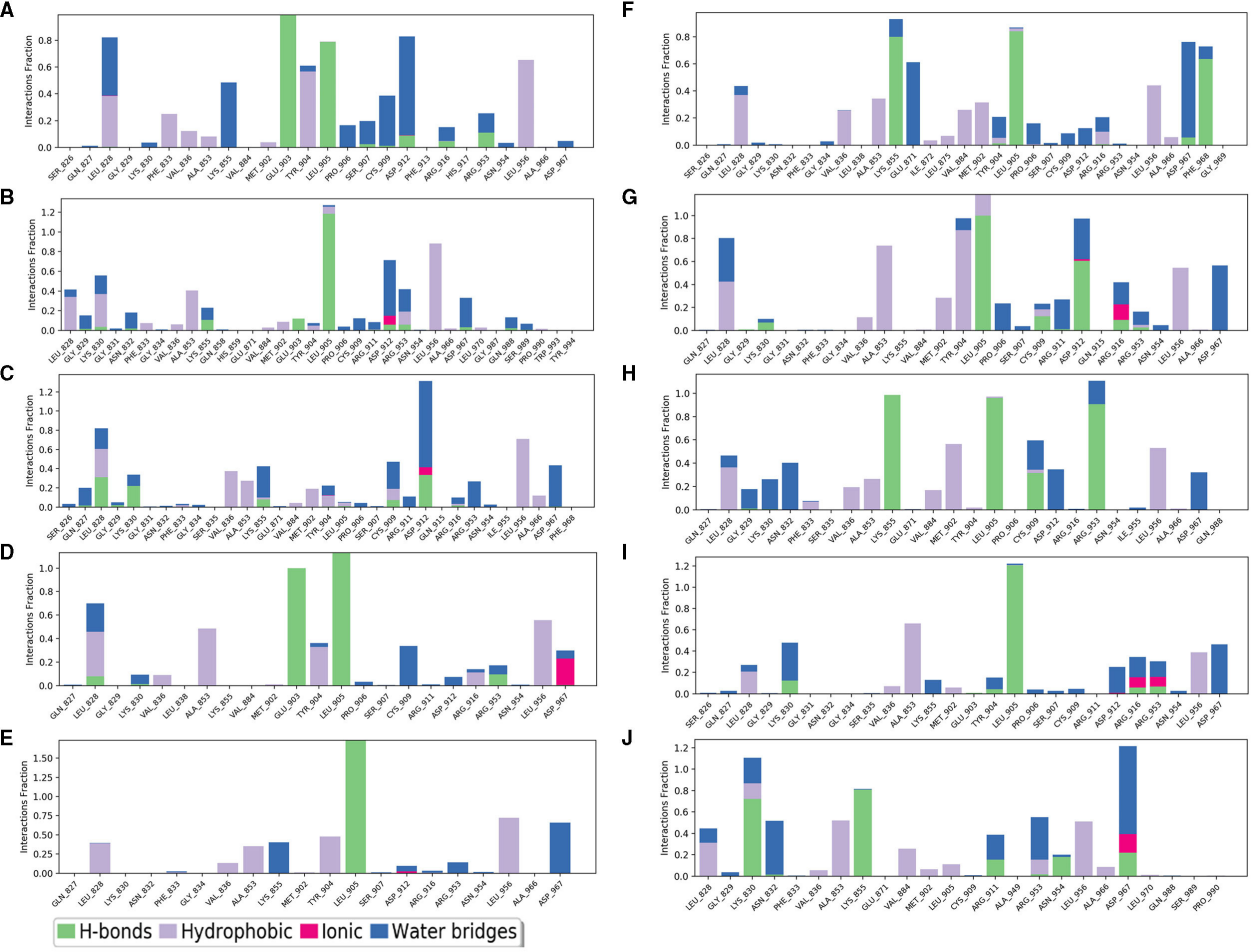
The contribution of the compound-JAK3 complex after MD simulation. **(A–J)** refers to compounds **1**–**10**.

The selected inhibitors underwent individual MD simulations for 1,000 ns, and their protein-ligand interactions were continually monitored as shown in Figure 6. H-bond interaction played a crucial role in ligand-protein binding and was important in drug design due to its significant impact on active compound design, metabolism, and adsorption. For the 10 molecules being selected above, compound **8** formed H-bond interactions with the amino acid residues Lys855 and Leu905 of the protein. Of note, the interaction fractions (ordinate) reached 0.6, suggesting the specific interactions were stably maintained for at least 60% of the simulation time, and meanwhile, it also elucidated the reason why compound **8** demonstrated strong inhibitory activity against the JAK3 and MOLM-16 cell lines (Figure 6). Compound 8 could have a strong H-bond interaction with Arg953 compared with other compounds in the JAK3 complex, indicating the importance of this specific residue for the ligand–JAK3 binding. Additionally, our previous calculations indicated that compound **8** possessed the third and second highest free energy as calculated by MMGBSA and DeepDock, respectively (Table 1). These results suggested that these two scoring methods in the JAK3 inhibitor discovery process might have higher evaluation efficacy.

## 4. Discussion and conclusion

As a druggable target, the JAK3 pathway has been studied widely, and some inhibitors are in clinical study (Figure 1) (2, 28). Importantly, JAK3 has also been confirmed as a target for the treatment of hematologic tumors (6). Therefore, it is meaningful to discover JAK3 inhibitors to treat cancer. Recently, virtual screening with artificial intelligence has become a popular method for drug discovery with low cost and high efficiency (29). Hence, we also introduced this combined strategy in our study.

The high expression or mutation of JAK3 is closely associated with cancer, especially hematologic tumors. Hence, the discovery of effective drugs targeting JAK3 is a hot area of research for the treatment of hematologic tumors and other related diseases. In this study, multiple HTVS methods were applied to identify molecules with high docking scores that could be evaluated for bioactivity. These methods included Glide_SP, Glide_XP, MM-GBSA, and DeepDock. Among the selected compounds, compound **8** exhibited inhibitory activity against JAK3 and MOLM-16 cells being tested *in vitro* (JAK3: 4,114 nM, MOLM-16 cell line: 1,834 nM). The precise binding mode between compound **8** and JAK3 was also characterized through MD simulations and indicated the importance of Arg953 for the ligand-JAK3 binding, which will serve as a reference for future structure optimization and drug development. Finally, this study also analyzed all 10 molecules through MD simulation, demonstrating the reason for compound **8** possessing better bioactivity against JAK3 compared with other compounds. This study identified a JAK3 inhibitor with a novel mother nucleus, providing a valuable foundation for the design and development of JAK3 inhibitors. Besides, our study also provided an appropriate “hit” for further structure modification to discover a new JAK3 inhibitor with high bioactivity and selectivity.

## Data availability statement

The original contributions presented in the study are included in the article/Supplementary material, further inquiries can be directed to the corresponding authors.

## Author contributions

WY, JW, ZS, and WH: methodology. YP and ZS: software and writing—review and editing. LX: validation. YP, LS, and ZS: formal analysis. YP: investigation. LS and ZS: resources and writing—original draft preparation. WH: data curation, supervision, and project administration. JW, YP, and ZS: visualization. WY and WH: funding acquisition. All authors have read and agreed to the published version of the manuscript.
