# Supplementary material for: A hybrid energy-based and AI-based screening approach for the discovery of novel inhibitors of JAK3
Source: Front Med (Lausanne). 2023 Oct 10;10:1182227. doi: 10.3389/fmed.2023.1182227 (PMC10598672; doi:10.3389/fmed.2023.1182227)
Supplement: Supplementary file 1 [file Data_Sheet_1.pdf]

A Hybrid Energy-based and AI-based Screening Approach for the Discovery of Novel Inhibitors of JAK3

Supplementary Material

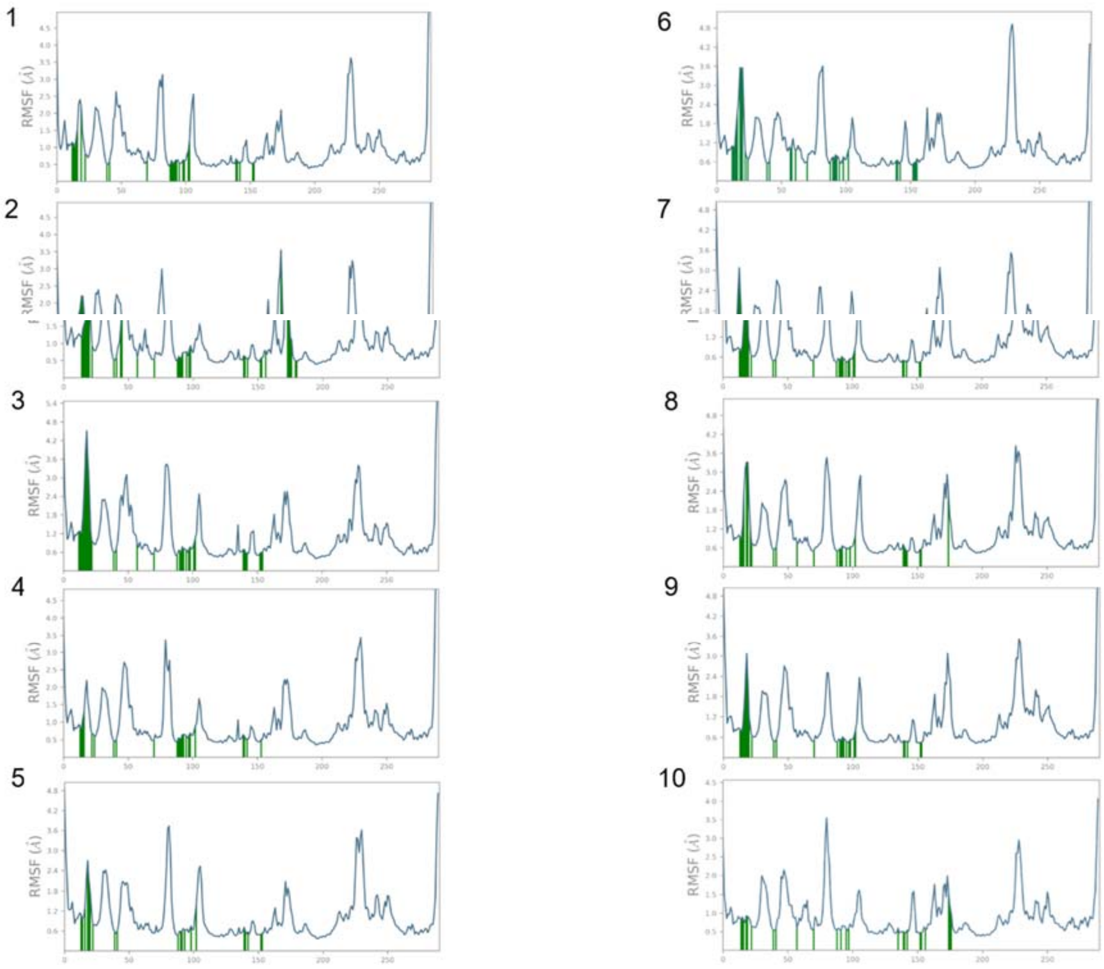

Figure S1. The RMSF after MD simulation. 1 - 10 refers to compound **1** - **10**.
